# Death of Dr. B. H. Catching

**Published:** 1899-12

**Authors:** 


### DEATH OF DR. B. H. CATCHING.

The announcement of the sudden death of Dr. Catching was received too late for an extended notice. This will be sad information to a wide professional circle, for his death will not only be a great loss to dentistry in the South, but will be equally felt throughout the profession. A full account of his life work will be given in the next number.

